# Plant-Derived Polyphenols Modulate Human Dendritic Cell Metabolism and Immune Function via AMPK-Dependent Induction of Heme Oxygenase-1

**DOI:** 10.3389/fimmu.2019.00345

**Published:** 2019-03-01

**Authors:** Nicole K. Campbell, Hannah K. Fitzgerald, Jean M. Fletcher, Aisling Dunne

**Affiliations:** School of Biochemistry and Immunology and School of Medicine, Trinity Biomedical Sciences Institute, Trinity College Dublin, The University of Dublin, Dublin, Ireland

**Keywords:** polyphenols, immunometabolism, dendritic cells, AMPK, HO-1 (heme oxygenase-1)

## Abstract

Polyphenols are important immunonutrients which have been investigated in the context of inflammatory and autoimmune disease due to their significant immunosuppressive properties. However, the mechanism of action of many polyphenols is unclear, particularly in human immune cells. The emerging field of immunometabolism has highlighted the significance of metabolic function in the regulation of immune cell activity, yet the effects of polyphenols on immune cell metabolic signaling and function has not been explored. We have investigated the effects of two plant-derived polyphenols, carnosol and curcumin, on the metabolism of primary human dendritic cells (DC). We report that human DC display an increase in glycolysis and spare respiratory capacity in response to LPS stimulation, which was attenuated by both carnosol and curcumin treatment. The regulation of DC metabolism by these polyphenols appeared to be mediated by their activation of the cellular energy sensor, AMP-activated Protein Kinase (AMPK), which resulted in the inhibition of mTOR signaling in LPS-stimulated DC. Previously we have reported that both carnosol and curcumin can regulate the maturation and function of human DC through upregulation of the immunomodulatory enzyme, Heme Oxygenase-1 (HO-1). Here we also demonstrate that the induction of HO-1 by polyphenols in human DC is dependent on their activation of AMPK. Moreover, pharmacological inhibition of AMPK was found to reverse the observed reduction of DC maturation by carnosol and curcumin. This study therefore describes a novel relationship between metabolic signaling via AMPK and HO-1 induction by carnosol and curcumin in human DC, and characterizes the effects of these polyphenols on DC immunometabolism for the first time. These results expand our understanding of the mechanism of action of carnosol and curcumin in human immune cells, and suggest that polyphenol supplementation may be useful to regulate the metabolism and function of immune cells in inflammatory and metabolic disease.

## Introduction

The emerging field of immunometabolism has highlighted the significance of metabolic function in the regulation of immune cell activity. Under certain conditions, anabolic and catabolic metabolism have become associated with pro- and anti-inflammatory immune responses, respectively (1). Thus, modulation of specific metabolic pathways in immune cells may represent a novel strategy to downregulate inflammation and promote the generation of anti-inflammatory immune responses. Polyphenols are a class of compound comprised of multiple phenol rings which naturally occur in plants, including fruits, vegetables, herbs and spices. Many polyphenols have been reported to exhibit significant anti-inflammatory activity and hold potential as immunonutrient supplements to treat inflammatory and autoimmune disease (2–8). However, the mechanism by which polyphenols regulate the immune response is unclear, and the relationship between immunonutrients and metabolism has been under-explored.

Dendritic cells (DC) play a central role in the generation of both innate and adaptive immune responses and it is now recognized that coordination of both immunological and metabolic signaling pathways is required for DC maturation. Murine bone marrow derived DC (BMDC) have been described to undergo a switch to Warburg metabolism upon activation, which is characterized by a strong upregulation of aerobic glycolysis via activation of the master growth/metabolic regulator, mammalian target of rapamycin (mTOR), and is accompanied by significant downregulation of oxidative phosphorylation (9–12). Conversely, this metabolic program is suppressed in immature BMDC by high activity of the cellular energy sensor, AMP-activated protein kinase (AMPK), which inhibits mTOR activation (10, 13). The downregulation of oxidative phosphorylation in BMDC during the switch to Warburg metabolism has been reported to result from suppression of mitochondrial activity by inducible nitric oxide synthase (iNOS)-derived NO (14). However, human DC and macrophages do not generally express iNOS (15–17), therefore, it is unclear whether they engage Warburg metabolism like their murine counterparts. A recent study by Malinarich et al. found that while mature human DC are more glycolytic than immature DC, they do not entirely downregulate oxidative phosphorylation, and instead display a more “balanced” switch to glycolysis (18). As the data available on the metabolic function of human DC is limited, it remains unclear to what extent human DC metabolism reflects that of murine DC.

Previous work from our laboratory has investigated the anti-inflammatory activity of the plant-derived polyphenols, carnosol and curcumin, in human DC. We reported that both of these polyphenols are capable of inhibiting DC maturation and maintain DC in a tolerogenic state through upregulation of the stress-response enzyme, heme oxygenase-1 (HO-1) (19). HO-1 is an important anti-inflammatory and antioxidant enzyme involved in heme/iron and redox metabolism, and its expression is strongly associated with the maturation status of DC (20–23). Despite its significant immunomodulatory effects in DC and established role in cellular metabolism, the relationship between HO-1 and DC immunometabolism has not yet been investigated. Furthermore, although certain polyphenols have been reported to activate AMPK in non-immune cells (24), it is unknown whether the anti-inflammatory effects of polyphenols, such as carnosol and curcumin, results from regulation of metabolic signaling.

In this study, we aimed to characterize the metabolic profile of human DC in response to LPS stimulation and to explore the effects of the polyphenols, carnosol and curcumin, on DC metabolism and downstream immune modulatory function. We report that, unlike BMDC, human DC stimulated with LPS upregulate both glycolysis and oxidative phosphorylation within hours of activation, however, the upregulation of glycolytic metabolism and spare respiratory capacity in maturing DC is inhibited by both carnosol and curcumin. We also demonstrate that both polyphenols strongly activate AMPK in human DC and effectively inhibit mTOR activation in response to LPS stimulation in an AMPK-dependent manner. Finally, we report that the upregulation of HO-1 by carnosol and curcumin, and consequential modulation of DC immune function, is dependent on their ability to activate AMPK. These findings enhance our understanding of DC immunometabolism and describe a novel relationship between AMPK, HO-1, and DC function which further underpins the anti-inflammatory activity of these plant-derived polyphenols.

## Materials and Methods

### Reagents

Carnosol (from *Rosemarinus officinalis*) and curcumin (from *Curcuma longa*) were purchased from Sigma-Aldrich and dissolved in DMSO. Ultrapure lipopolysaccharide (LPS) from *E. coli* serotype O111:B4 was purchased from Enzo Life Sciences. The AMPK inhibitor compound C (also known as dorsomorphin) was purchased from Sigma-Aldrich and dissolved in DMSO. The AMPK agonist 5-Aminoimidazole-4-carboxamide ribonucleotide (AICAR) was purchased from Sigma-Aldrich and dissolved in water.

### Human Blood Samples

This study was approved by the research ethics committee of the School of Biochemistry and Immunology, Trinity College Dublin and was conducted in accordance with the Declaration of Helsinki. Leukocyte-enriched buffy coats from anonymous healthy donors were obtained with permission from the Irish Blood Transfusion Service (IBTS), St. James's Hospital, Dublin. Donors provided informed written consent to the IBTS for their blood to be used for research purposes. PBMC were isolated by density gradient centrifugation (Lymphoprep; Axis-Shield poC). Cells were cultured in RPMI medium supplemented with 10% FCS, 2 mM L-glutamine, 100 U/ml penicillin and 100 μg/ml streptomycin (all Sigma Aldrich) and maintained in humidified incubators at 37°C with 5% CO_2_.

### Dendritic Cell Culture

CD14^+^ monocytes were positively selected from PBMC by magnetic sorting using a MagniSort Human CD14 Positive Selection kit (eBioscience) according to the manufacturer's protocol. Monocyte-derived DC were produced by culturing purified CD14^+^ monocytes at 1 × 10^6^ cells/ml in complete RPMI supplemented with GM-CSF (50 ng/ml) and IL-4 (40 ng/ml; both Miltenyi Biotec). On the third day of culture half the media was removed and replaced with fresh media supplemented with cytokines. After 6 days non-adherent and loosely adherent cells were gently removed. The purity of CD14^lo^DC-SIGN^+^ DC was assessed by flow cytometry and was routinely >98%.

### Western Blotting

For detection of AMPK expression, DC were cultured at 1 × 10^6^ cells/ml in the presence of AICAR (1 mM), carnosol (10 μM), curcumin (10 μM) or a vehicle control (DMSO), for 1 h. For detection of HO-1 expression, DC were cultured at 1 × 10^6^ cells/ml with AICAR (125–1,000 μM) for 24 h, or with compound C (5 μM) for 1 h prior to incubation with carnosol (10 μM), curcumin (10 μM) or DMSO for 24 h. For detection of pS6 expression, DC were cultured at 1 × 10^6^ cells/ml with compound C (5 μM) for 1 h prior to incubation with carnosol (10 μM), curcumin (10 μM) or DMSO for 1 h, followed by stimulation with LPS (100 ng/ml) for 1 h. Cell lysates were prepared by washing cells in PBS prior to lysis in RIPA buffer (Tris 50 mM; NaCl 150 mM; SDS 0.1%; Na.Deoxycholate 0.5%; Triton X 100) containing phosphatase inhibitor cocktail set (Sigma-Aldrich). Samples were electrophoresed and transferred to PVDF prior to incubation with monoclonal antibodies specific for HO-1 (Enzo Life Sciences), ribosomal protein S6 phosphorylated at Ser235 and Ser236, AMPK phosphorylated at Thr172, and total AMPK (all Cell Signaling), overnight at 4°C. Membranes were then washed in TBS-Tween and incubated with anti-rabbit streptavidin-conjugated secondary antibody (Sigma Aldrich) for 2 h at room temperature, prior to development with enhanced chemiluminescent substrate (Merck Millipore) using a BioRad ChemiDoc MP system. Subsequently, membranes were re-probed with HRP-conjugated monoclonal antibodies specific for β-actin (Sigma-Aldrich) as a loading control. Full length blots are presented in Supplementary Figures 1, 2.

### Flow Cytometry

DC were cultured at 1 × 10^6^ cells/ml in the presence of compound C (5 μM) for 1 h, followed by incubation with carnosol (10 μM), curcumin (10 μM) or DMSO for 6 h prior to stimulation with LPS (100 ng/ml). After 24 h, DC were removed for analysis by flow cytometry. DC were collected, washed in PBS and stained extracellularly with amine-binding markers for dead cells (Fixable Viability Dye; eBioscience) and fluorochrome-conjugated antibodies for CD40 (Invitrogen), CD80, CD83, and CD86 (all Biolegend). For phagocytosis assays, DC were cultured with complete RPMI containing DQ-Ovalbumin (500 ng/ml; Invitrogen) for 20 min at 37°C, followed by incubation for 10 min at 4°C. DC were then washed in PBS and immediately acquired. Acquisition was performed on either a BD FACS Canto II or LSR Fortessa, and analysis was performed with FlowJo v.10 software (Tree Star Inc.). Gating strategies are presented in Supplementary Figure 3.

### Seahorse Analyser

Due to limitations in cell numbers, the glycolytic and respiratory profiles of DC were measured simultaneously using a combined glycolysis/mitochondrial stress test, as previously described (25). This includes the addition of pyruvate in the cell culture media in order to determine the basal respiratory rate of the cells. While this approach can artificially result in increased glycolysis measurements, as the same media was used for all treatment groups this artifact does not alter the internal validity of the obtained results.

DC were cultured at 2 × 10^5^ cells/well in a Seahorse 24-well microplate. The Seahorse cartridge plate was hydrated prior to use by the addition of 1 ml XF calibrant fluid per well and incubated in a non-CO_2_ incubator at 37°C for a minimum of 8 h prior to use. To measure DC metabolism in response to LPS stimulation, DC were stimulated with LPS (100 ng/ml) for 1, 3, 6 or 24 h prior to analysis using a Seahorse XF24 analyser. To determine the effect of polyphenols on DC metabolism, DC were incubated with carnosol (10 μM), curcumin (10 μM), or DMSO for 6 h, followed by stimulation with LPS (100 ng/ml) for 6 h, prior to analysis using a Seahorse XFe24 analyser. Between 30 and 60 min prior to placement into the XF/XFe analyser, cell culture medium was replaced with complete XF assay medium (Seahorse Biosciences; supplemented with 10 mM glucose, 1 mM sodium pyruvate, 2 mM L-glutamine, and pH adjusted to 7.4) and incubated in a non-CO_2_ incubator at 37°C. Blank wells (XF assay medium only) were prepared without cells for subtracting the background oxygen consumption rate (OCR) and extracellular acidification rate (ECAR) during analysis. Oligomycin (1 μg/ml; Cayman Chemicals), carbonyl cyanide-p-trifluoromethoxyphenylhydrazone (FCCP) (450 nM; Santa Cruz biotechnology), rotenone (500 nM), antimycin A (2.5 μM), and 2-deoxy-D-glucose (2-DG) (25 mM; all Sigma-Aldrich) were prepared in XF assay medium and loaded into the appropriate injection ports on the cartridge plate (75 μl/port) and incubated for 10 min in a non-CO_2_ incubator at 37°C. The cartridge was then placed into the XF/XFe analyser and the machine was calibrated. Following calibration the cell plate was added to the XF/XFe analyser and the OCR and ECAR were measured over time with sequential injections of (A) oligomycin, (B) FCCP, (C) rotenone and antimycin A, and (D) 2-DG. Upon completion of the assay the XF assay medium was removed and RIPA buffer was added to each well. Protein concentration was determined by the Pierce BCA assay (ThermoFisher) to ensure protein content was similar between all treatment wells. Analysis of results was performed using Wave software (Agilent Technologies). The rates of basal glycolysis, max glycolysis, glycolytic reserve, basal respiration, max respiration and respiratory reserve were calculated as detailed in Table 1.

**Table 1:** Seahorse calculations.

**Rate**	**Calculation**
Non–glycolytic ECAR	Average ECAR values after 2–DG treatment.
Basal glycolysis	Average ECAR values prior to oligomycin treatment–non–glycolytic ECAR
Max glycolysis	Average ECAR values after oligomycin & before FCCP treatment
Glycolytic reserve	Max glycolysis–basal glycolysis
Basal respiration	Average OCR values prior to oligomycin treatment–non–mitochondrial OCR
Max respiration	Average OCR values after FCCP & before rotenone/antimycin A treatment
Respiratory reserve	Max respiration–basal respiration

### Statistical Analysis

Statistical analysis was performed using Prism 6 software (GraphPad Software Inc.). Analysis of 3 or more data sets was performed by repeated measures one-way ANOVA with either Tukey's, Dunnett's or Sidak's *post hoc* test as appropriate; *p*-values < 0.05 were considered significant and are denoted with asterisks in the figures.

## Results

### Human DC Temporally Upregulate Glycolysis and Oxidative Phosphorylation After LPS Stimulation

The current understanding of DC metabolism is largely based on murine studies, which have demonstrated that BMDC strongly upregulate aerobic glycolysis and downregulate oxidative phosphorylation upon TLR stimulation (10–12). However, this engagement of Warburg metabolism has been reported to be dependent on NO produced by iNOS in BMDC (14). Human monocyte-derived DC do not typically express iNOS, and therefore it is likely that their metabolic function may differ from that of BMDC (17). A recent study investigating the metabolism of tolerogenic vs. immunogenic human DC confirmed that LPS-matured DC do not undergo a switch to Warburg metabolism (18). However, the metabolism of human DC was only assessed 24 h after stimulation. As the metabolic changes of BMDC have been observed to occur rapidly after TLR stimulation (12), it was of interest in the present study to first characterize the metabolic changes of LPS-stimulated human DC over time. Human DC were seeded into a Seahorse microplate and stimulated with LPS for 0, 1, 3, 6, or 24 h prior to placement into a Seahorse XF24 analyser. The rate of glycolysis and oxidative phosphorylation were determined by the measured ECAR and OCR, respectively, after addition of oligomycin (an inhibitor of mitochondrial complex V), FCCP (a mitochondrial uncoupler), rotenone and antimycin A (inhibitors of the mitochondrial complexes I & III, respectively), and 2-DG (an inhibitor of glycolysis).

**Figure 1:**
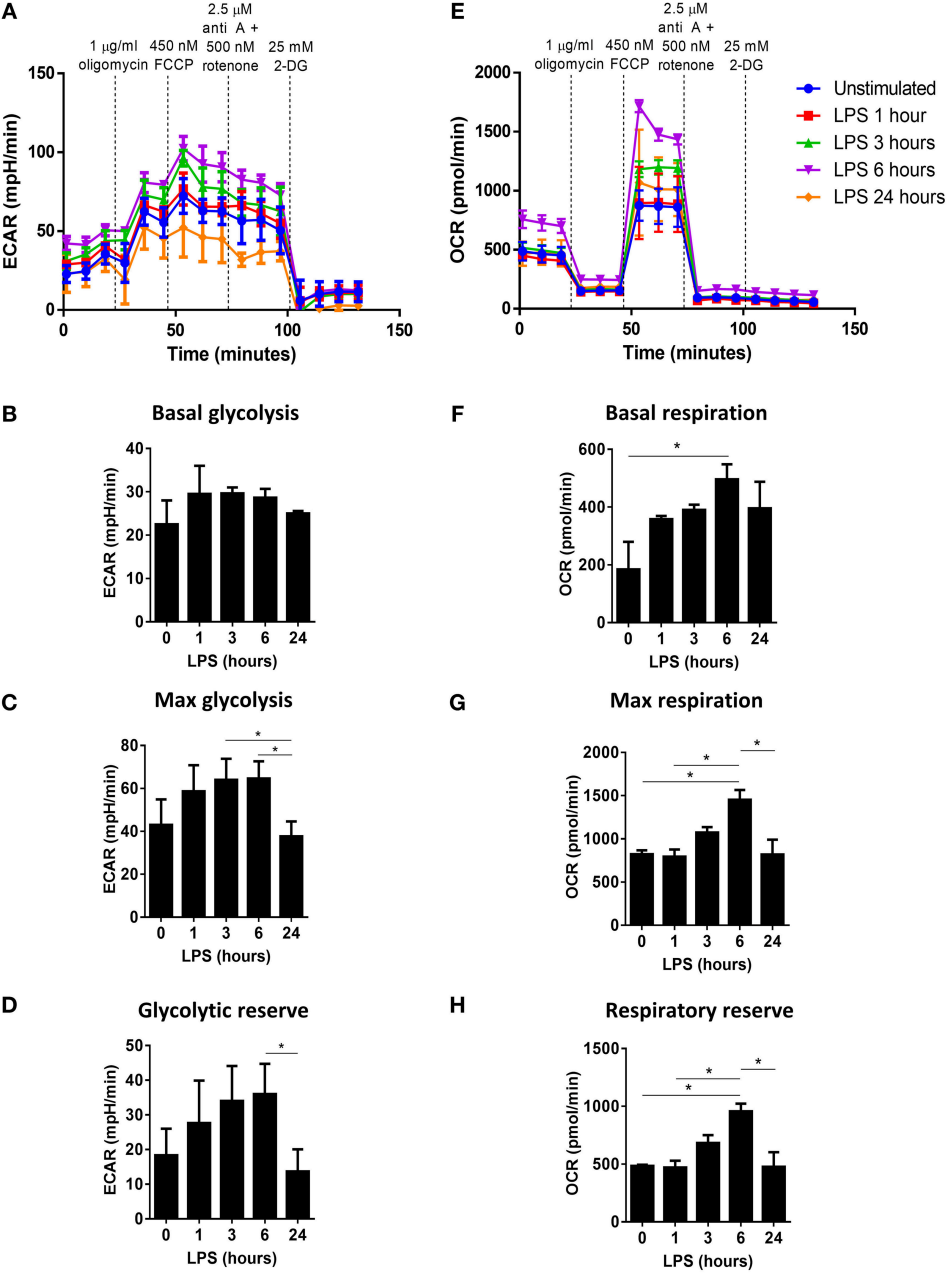
Determination of the changes in glycolytic metabolism and oxidative phosphorylation over time in LPS-stimulated human DC. Primary human DC (*n* = 3) were stimulated with LPS (100 ng/ml) for 1, 3, 6, or 24 h prior to placement in a Seahorse XF24 analyser. The extracellular acidification rate (ECAR) and oxygen consumption rate (OCR) were measured before and after the addition of oligomycin (1 μg/ml), FCCP (450 nM), antimycin A (2.5 μM), and rotenone (500 nM), and 2-DG (25 mM). **(A)** ECAR measurements over time for each LPS stimulation time-point. Data depicts one representative experiment. Pooled data (*n* = 3) depicts the calculated mean (± SEM) **(B)** basal glycolytic rate, **(C)** max glycolytic rate, and **(D)** glycolytic reserve for each LPS stimulation time-point. **(E)** OCR measurements over time for each LPS stimulation time-point. Data depicts one representative experiment. Pooled data (*n* = 3) depicts the calculated mean (± SEM) **(F)** basal respiratory rate, **(G)** max respiratory rate, and **(H)** respiratory reserve for each LPS stimulation time-point. Statistical significance was determined by repeated measures one-way ANOVA with Tukey's multiple comparisons *post hoc* test to compare the means of all treatment groups (^*^p < 0.05).

The ECAR of LPS-stimulated DC was highest at 3 and 6 h post-LPS treatment, while the ECAR of DC 24 h post-LPS treatment was observed to be similar to that of unstimulated DC (Figure 1A). The glycolytic profile of unstimulated vs. LPS-stimulated DC was assessed, and it was observed that the basal rate of glycolysis was increased in LPS-treated DC at all timepoints (Figure 1B). However, the maximum rate of glycolysis increased in LPS-stimulated DC after 1 h, and peaked at 3–6 h before returning to the unstimulated-DC baseline by 24 h post-LPS (Figure 1C). This was reflected in the calculated glycolytic reserve of LPS-stimulated DC, which was greatest in DC 3–6 h post-LPS, whereas at 24 h post-LPS stimulation, DC displayed a glycolytic reserve similar to unstimulated DC (Figure 1D). Therefore, while stimulation of human DC with LPS results in a small but mostly sustained increase in the basal glycolytic rate, the increased glycolytic potential of LPS-stimulated DC appears to be transient, peaking at 3–6 h post-activation. Furthermore, the respiratory profiles of DC appeared to mirror their observed glycolytic activity; DC stimulated with LPS for 6 h displayed the highest OCR, while smaller increases in the OCR of DC 1, 3, and 24 h post-LPS were seen compared to unstimulated DC (Figure 1E). The basal respiratory rate of LPS-stimulated DC was higher than that of unstimulated DC at all timepoints, and was significantly increased in DC 6 h post-LPS treatment (Figure 1F). Interestingly, the maximal respiratory rate (Figure 1G) and respiratory reserve (Figure 1H) were significantly increased in DC stimulated with LPS for 6 h compared to both unstimulated DC and DC treated with LPS for 1 or 24 h. Taken together, these data indicate that, unlike murine DC, human DC upregulate both glycolytic metabolism and oxidative phosphorylation upon LPS-stimulation. However, this observed increase in DC metabolism peaks approximately 6 h post-activation.

### The Plant-Derived Polyphenols, Carnosol and Curcumin, Inhibit the Metabolic Reprogramming of Human DC in Response to LPS Stimulation

Human DC were observed to undergo significant metabolic reprogramming during LPS stimulation, characterized by an increased basal rate of glycolysis and oxidative phosphorylation, and a temporary increase in glycolytic and respiratory capacity. We have previously reported that the plant-derived polyphenols, carnosol and curcumin, inhibit the maturation and immune function of human DC (19). Given that upregulation of cellular metabolism has been reported to be essential for BMDC maturation (10–12), it was of interest to investigate whether treatment with carnosol and curcumin might alter the metabolic reprogramming observed in human DC upon stimulation with LPS. As the greatest upregulation of glycolysis and oxidative phosphorylation was seen at 6 h post LPS stimulation, this timepoint was chosen to assess the action of carnosol and curcumin on DC metabolism. Human DC were seeded into a Seahorse microplate and treated with carnosol or curcumin for 6 h prior to stimulation with LPS for a further 6 h. DC were then placed into a Seahorse XFe24 analyser and their metabolic activity was determined by the measured ECAR & OCR in response to metabolic inhibitors, as described before.

**Figure 2:**
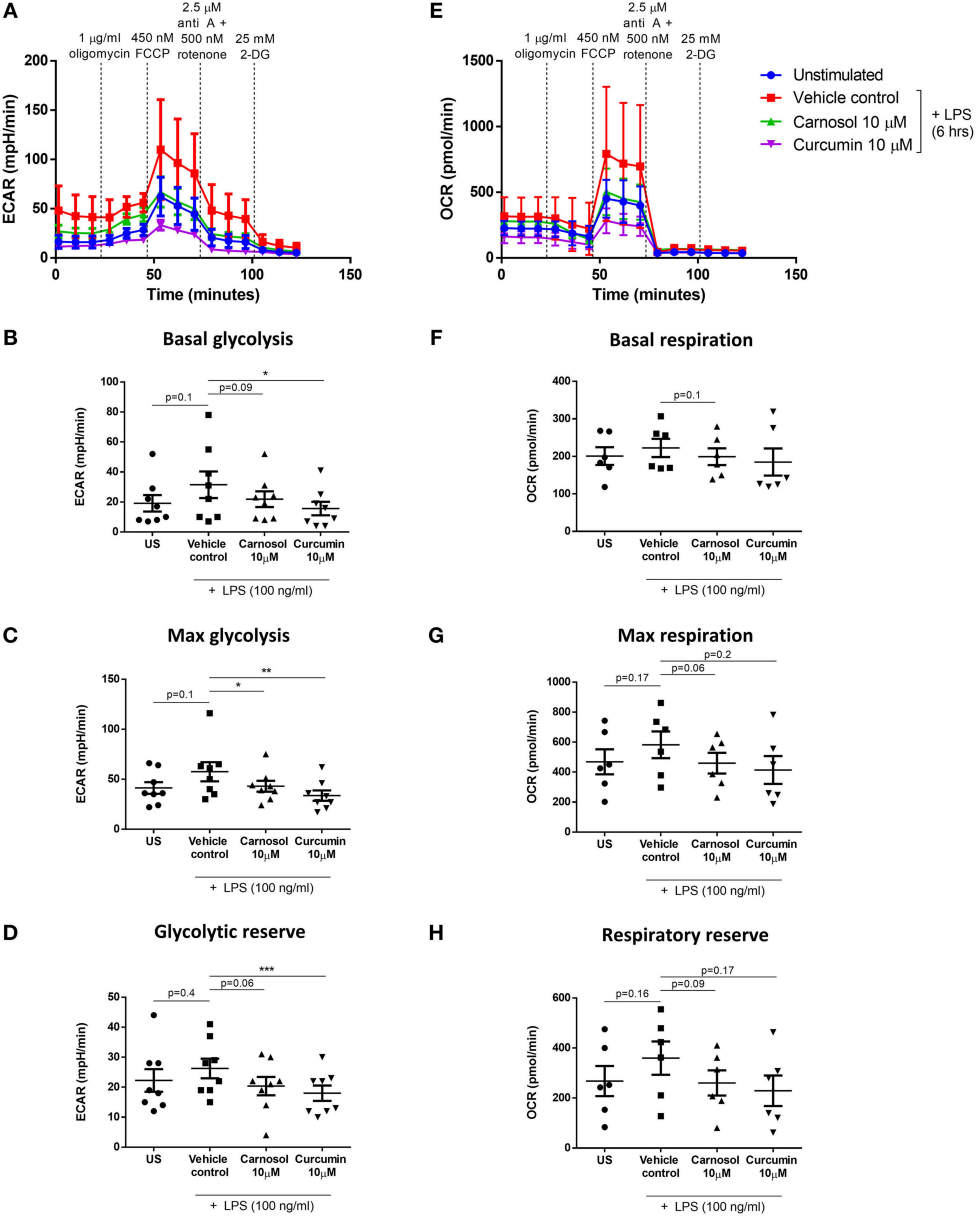
Carnosol and curcumin reduce the upregulation of glycolysis and spare respiratory capacity of LPS-stimulated DC. Primary human DC were either left unstimulated (US), or treated with carnosol (10 μM), curcumin (10 μM) or a vehicle control for 6 h, then stimulated with LPS (100 ng/ml) for 6 h prior to placement in a Seahorse XFe24 analyser. The extracellular acidification rate (ECAR) and the oxygen consumption rate (OCR) were measured before and after the addition of oligomycin (1 μg/ml), FCCP (450 nM), antimycin A (2.5 μM), and rotenone (500 nM), and 2-DG (25 mM). **(A)** ECAR measurements over time for each treatment group. Data depicts one representative experiment. Pooled data (*n* = 8) depicts the calculated mean (± SEM) **(B)** basal glycolytic rate, **(C)** max glycolytic rate and **(D)** glycolytic reserve for each treatment group. **(E)** OCR measurements over time for each treatment group. Data depicts one representative experiment. Pooled data (*n* = 6) depicts the calculated mean (± SEM) **(F)** basal respiratory rate, **(G)** max respiratory rate, and **(H)** respiratory reserve for each treatment group. Statistical significance was determined by repeated measures one-way ANOVA with Dunnett's multiple comparisons *post hoc* test to compare treatment groups against the control group (^***^p < 0.001, ^**^p < 0.01, ^*^p < 0.05).

As previously observed, the ECAR of LPS-stimulated DC was higher than that of unstimulated DC, whereas LPS-stimulated DC previously treated with either carnosol or curcumin displayed an ECAR similar to unstimulated DC (Figure 2A). This was reflected in the basal rate of glycolysis, which was significantly reduced in curcumin-treated DC compared to control DC, and a trend toward reduced basal glycolysis was also seen in carnosol-treated DC (Figure 2B). The observed inhibition of glycolysis in carnosol- and curcumin-treated DC was more pronounced in the maximal rate of glycolysis (Figure 2C) and glycolytic reserve (Figure 2D), which were significantly reduced with both polyphenols compared to control DC. The OCR of LPS-stimulated DC was also observed to be greater than that of unstimulated DC, and of carnosol- and curcumin-treated DC (Figure 2E). A slight reduction in the basal respiratory rate was observed in carnosol and curcumin treated DC compared to control DC, but this was not significant (Figure 2F). Conversely, a trend toward an increased maximal respiratory rate (Figure 2G) and respiratory reserve (Figure 2H) was observed in LPS-stimulated DC compared to unstimulated DC, which was reduced in DC previously treated with carnosol or curcumin.

### Carnosol and Curcumin Inhibit mTOR Activity and Upregulate HO-1 Expression in Human DC via Activation of AMPK

**Figure 3:**
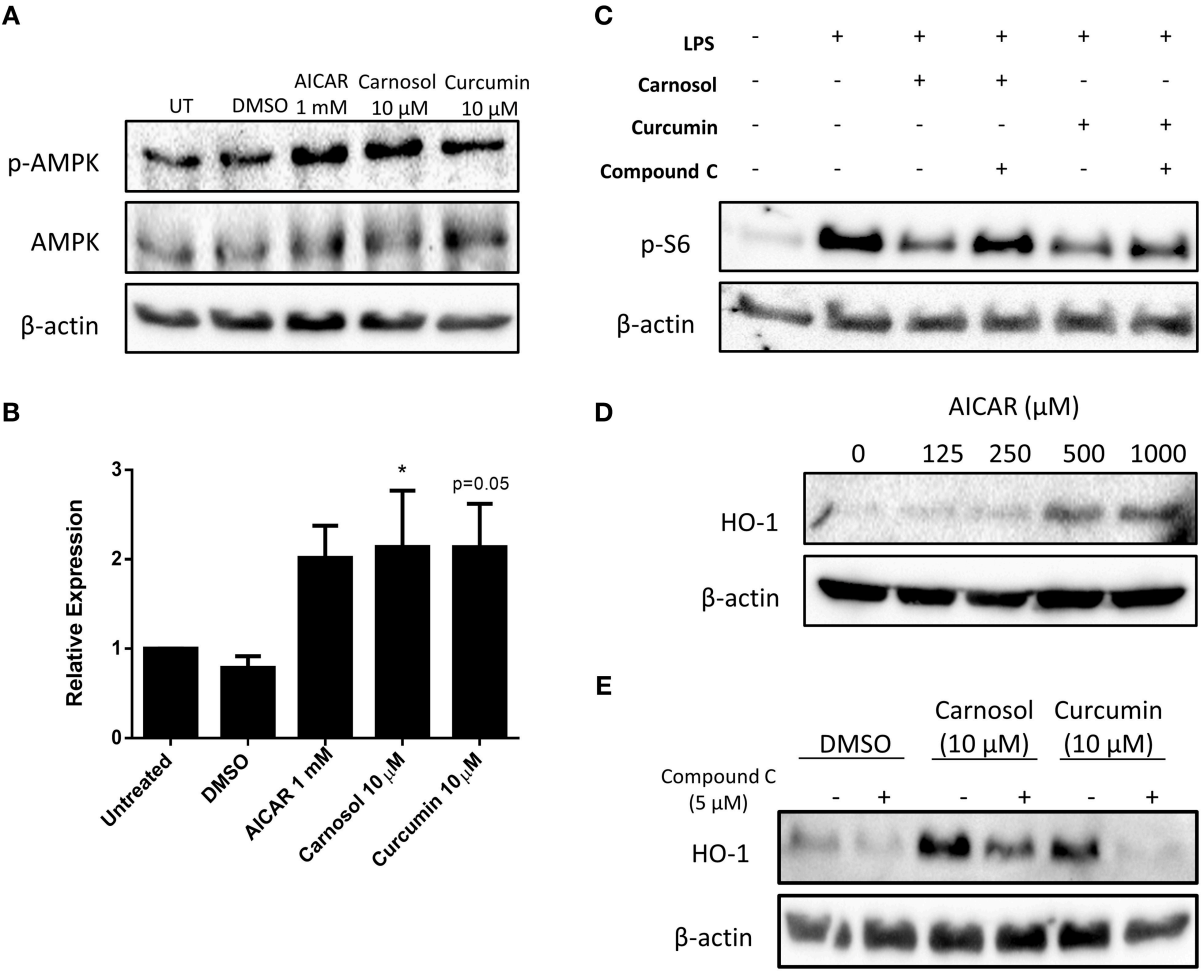
Carnosol and curcumin inhibit mTOR activity and upregulate HO-1 expression in human DC via activation of AMPK. **(A)** Primary human DC were incubated with AICAR (1 mM), carnosol (10 μM), curcumin (10 μM), or a vehicle control for 1 h. Activation of AMPK was measured by Western blot. **(B)** Pooled data (*n* = 7) depicting densitometric analysis of phospho-AMPK expression relative to the loading control. **(C)** Primary human DC were incubated with compound C (5 μM) for 1 h prior to treatment with carnosol (10 μM) or curcumin (10 μM) for 1 h, followed by stimulation with LPS (100 ng/ml) for 1 h. Expression of phospho-S6 was determined by Western blot. **(D)** Primary human DC were incubated with AICAR (125–1,000 μM) for 24 h. Expression of HO-1 was detected by Western blot. **(E)** Primary human DC were incubated with compound C (5 μM) for 1 h prior to treatment with carnosol (10 μM) or curcumin (10 μM), or a vehicle control for 24 h. Expression of HO-1 was detected by Western blot. All blots depict an individual donor and are representative of 3–7 independent experiments. Blots shown are derived from the same gel(s); membranes were first probed for the protein of interest and then re-probed for β-actin as a loading control. Full-length blots are presented in Supplementary Figures 1, 2. Statistical significance was determined by one-way ANOVA with Dunnett's multiple comparisons *post hoc* test to compare treatment groups against the control group (^*^p < 0.05).

Previous work from our laboratory has demonstrated that carnosol and curcumin exert extensive immunomodulatory and anti-inflammatory effects in human DC as a result of their upregulation of HO-1 expression (19). The cellular energy sensor and master regulator of catabolic metabolism, AMPK, has been described to suppress glycolytic metabolism and pro-inflammatory responses in BMDC (10, 13). Furthermore, AMPK has been implicated in the induction of HO-1 expression in other cell types (26–28). Thus, it was hypothesized that signaling via AMPK may regulate the inhibition of DC metabolism and induction of HO-1 by the polyphenols carnosol and curcumin. To determine whether carnosol or curcumin treatment results in activation of AMPK in human DC, DC were treated with carnosol, curcumin, or AICAR, an AMPK agonist, for 1 h. Phosphorylation, and therefore activation, of AMPK was detected by Western blot. Treatment with AICAR, carnosol and curcumin were all found to increase the activation of AMPK compared to control DC (Figures 3A,B).

One of the primary mechanisms by which AMPK regulates cellular metabolism is through inhibition of mTOR, the major promoter of anabolic metabolism which is highly activated in response to LPS stimulation (29–31). Having confirmed that carnosol and curcumin can activate AMPK, it was next investigated whether they might inhibit mTOR activity in LPS-stimulated DC. The ribosomal protein S6 is phosphorylated downstream of mTOR activation and serves as a readout of mTOR activity. DC were treated with compound C, a pharmacological inhibitor of AMPK, for 1 h prior to incubation with carnosol or curcumin for 1 h, followed by stimulation with LPS for 1 h. The expression of phospho-S6 was detected by Western blot. As expected, stimulation of human DC with LPS resulted in a strong increase in phospho-S6 expression, which was attenuated in DC treated with either carnosol or curcumin. However, the reduction of phospho-S6 expression by carnosol and curcumin was reversed with the addition of compound C (Figure 3C).

Although AMPK signaling has been reported to regulate HO-1 expression in other cell types, there have been no reports of AMPK-dependent upregulation of HO-1 in DC. Therefore, to determine whether AMPK activation can upregulate HO-1 expression in human DC, DC were treated with increasing concentrations of AICAR for 24 h, after which the expression of HO-1 was detected by Western blot. A dose-dependent increase of HO-1 expression was observed in AICAR-treated DC, with the greatest upregulation observed at 0.5 mM and 1 mM (Figure 3D). Following this, the contribution of AMPK to the upregulation of HO-1 by carnosol and curcumin was investigated. DC were treated with compound C for 1 h prior to treatment with carnosol or curcumin. After 24 h, the expression of HO-1 was detected by Western blot. As previously observed (19), carnosol and curcumin increased the expression of HO-1 by DC, however, this increase was diminished in the presence of compound C (Figure 3E).

### Inhibition of AMPK Attenuates the Reduction of DC Maturation by Carnosol and Curcumin

**Figure 4:**
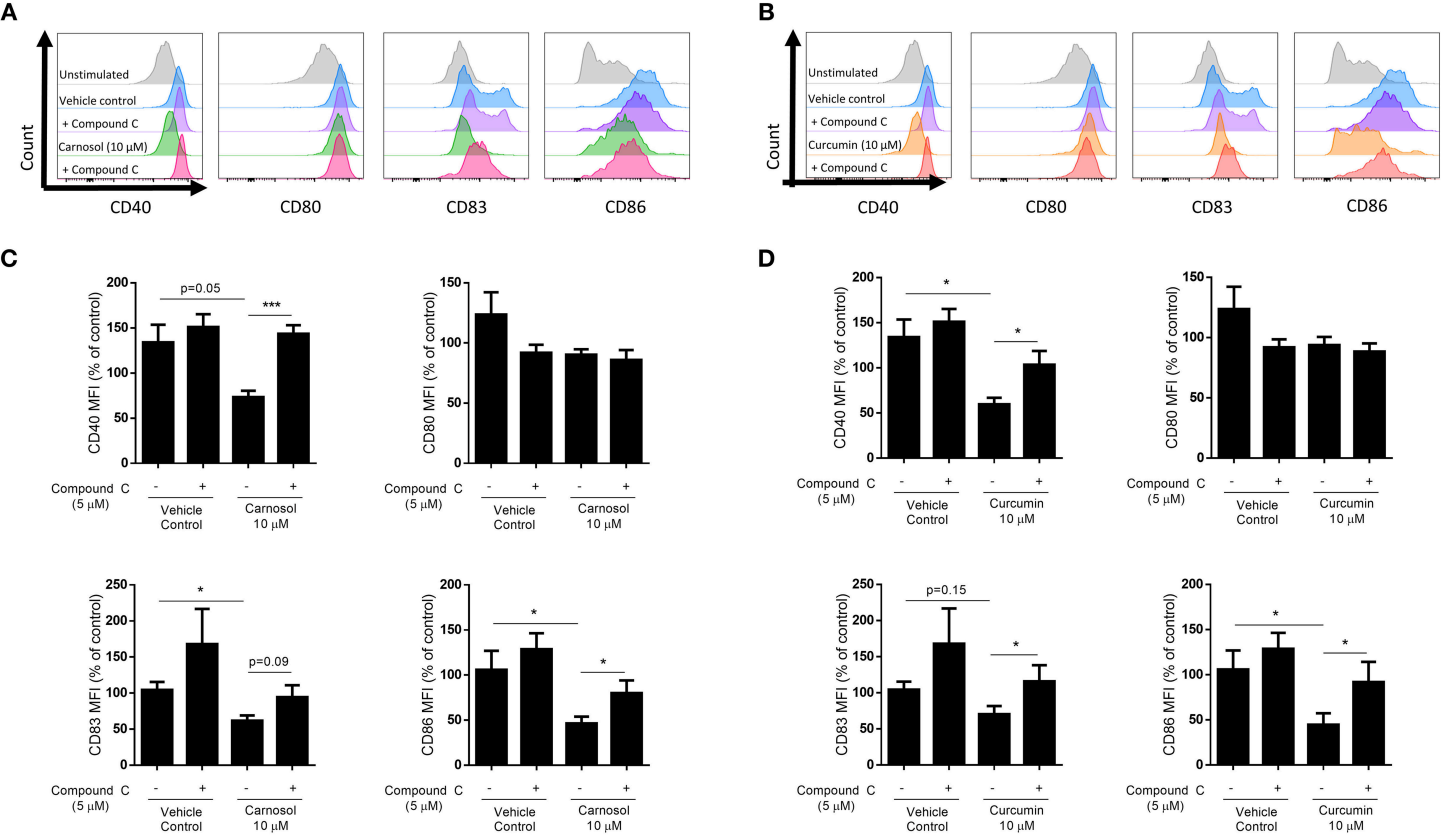
Inhibition of AMPK attenuates reduction of DC maturation markers by carnosol and curcumin. Primary human DC (*n* = 7) were incubated with or without compound C (5 μM) for 1 h prior to treatment with carnosol (10 μM), curcumin (10 μM), or a vehicle control for 6 h. DC were then stimulated with LPS (100 ng/ml) and expression of maturation markers CD40, CD80, CD83, and CD86 was determined after 24 h by flow cytometry. Histograms depict expression of maturation markers in DC treated with **(A)** carnosol or **(B)** curcumin, with or without compound C, compared to controls from one representative experiment. Pooled data (*n* = 7) depicts expression of CD40, CD80, CD83, and CD86 in DC treated with **(C)** carnosol or **(D)** curcumin, with or without compound C. Results shown are mean (± SEM) of the measured Mean Fluorescence Intensities (MFI), expressed as percentages of the vehicle control. Statistical significance was determined by repeated measures one-way ANOVA, with Sidak's multiple comparisons *post hoc* test to compare pre-selected group pairs (^***^p < 0.001, ^*^p < 0.05).

HO-1 is a known promoter of tolerogenic DC, as it is highly expressed in immature DC and limits their maturation in response to pro-inflammatory stimuli (21–23). Upregulation of HO-1 by carnosol and curcumin was previously observed to limit the maturation of human DC stimulated with LPS (19). As inhibition of AMPK via compound C was found to attenuate the induction of HO-1 by both carnosol and curcumin, it was next investigated whether AMPK inhibition could also reverse the effects of these polyphenols on DC maturation. Human DC were treated with compound C for 1 h before addition of either carnosol or curcumin for a further 6 h (to allow for the upregulation of HO-1 gene transcription and protein translation) prior to stimulation with LPS. After 24 h, expression of the maturation markers CD40 and CD83, and co-stimulatory molecules CD80 and CD86 was measured by flow cytometry. Consistent with previous observations (19), carnosol treatment significantly reduced expression of CD83 and CD86 by LPS-stimulated DC, with a trend toward reduced CD40 also observed. However, this effect was attenuated in the presence of compound C (Figures 4A,C). Similarly, curcumin treatment significantly reduced the expression of CD40 and CD86 in LPS stimulated DC, with a trend toward reduced CD83 also observed. Again, this inhibition of surface marker expression by curcumin was reversed with the addition of compound C (Figures 4B,D). Treatment of LPS-stimulated DC with compound C alone did not increase the expression of DC surface markers.

**Figure 5:**
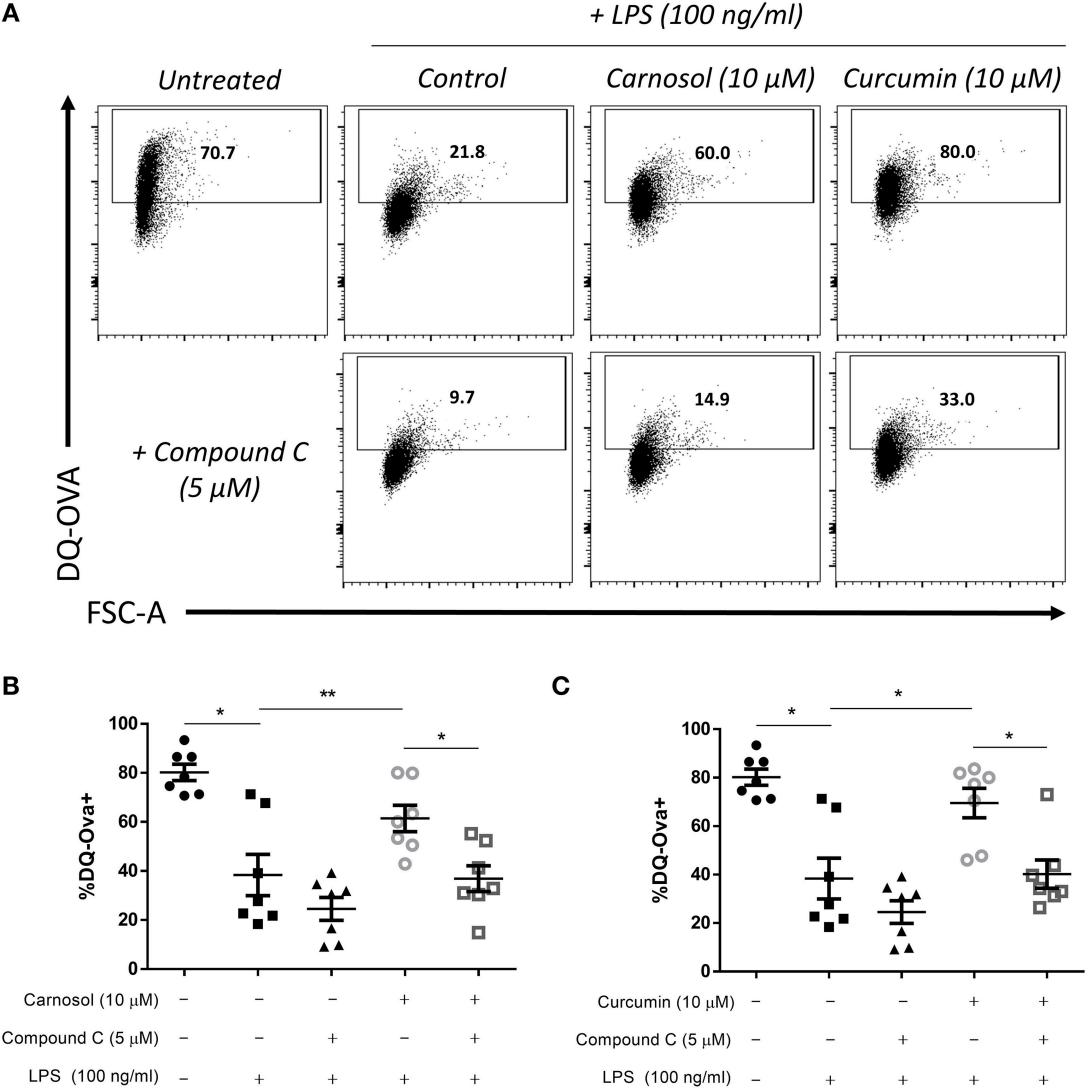
Inhibition of AMPK attenuates the increased phagocytic capacity of LPS-stimulated DC treated with carnosol or curcumin. Primary human DC (*n* = 7) were incubated with or without compound C (5 μM) for 1 h, then treated with carnosol (10 μM), curcumin (10 μM) or a vehicle control for 6 h prior to stimulation with LPS (100 ng/ml) for 24 h. DC were then incubated with DQ-Ovalbumin (DQ-Ova; 500 ng/ml) for 20 min prior to analysis by flow cytometry. **(A)** Representative dot plots depicting DQ-Ova uptake by DC treated with carnosol, curcumin, and compound C from one representative experiment. Pooled data (*n* = 7) depicts percentage DQ-Ova uptake of DC treated with **(B)** carnosol or **(C)** curcumin, with or without compound C. Results shown are mean (± SEM) percentages of DQ-Ova uptake in control-, carnosol- and curcumin-treated DC, with or without compound C. Statistical significance was determined by repeated measures one-way ANOVA, with Tukey's multiple comparisons *post hoc* test to compare means of all groups (^**^p < 0.01, ^*^p < 0.05).

In addition to increased expression of maturation and co-stimulatory markers, DC lose their capacity to take up/phagocytose antigens upon maturation as their role switches from tissue surveillance to antigen presentation (32). We have previously reported that treatment of human DC with carnosol or curcumin can maintain the capacity of DC to take up and process antigens after stimulation with LPS (19). Following the observation that inhibition of AMPK signaling via compound C reversed the effects of carnosol and curcumin on the phenotypic maturation of DC, it was next determined whether their effects on functional DC maturation would also be attenuated. DC were treated with compound C, carnosol or curcumin, and stimulated with LPS as before. After 24 h, DC were incubated with the model antigen DQ-Ovalbumin (DQ-Ova) for 20 min, and analyzed for antigen uptake by flow cytometry. As expected, stimulation of DC with LPS dramatically reduced their capacity to uptake antigen compared to immature DC. Furthermore, both carnosol and curcumin treatment maintained the phagocytic capacity of LPS-stimulated DC similar to that of immature DC (Figure 5A), as was observed previously (19). However, addition of compound C to carnosol- and curcumin-treated DC significantly abrogated this effect (Figures 5B,C). Treatment of DC with compound C alone did not significantly alter their antigen uptake capacity following LPS stimulation. Taken together, these results confirm that the immunomodulatory effects of carnosol and curcumin on both phenotypic and functional DC maturation are dependent on their activation of AMPK.

## Discussion

Supplementation with immunonutrients such as polyphenols represents a novel strategy to modulate the immune response through dietary intervention. However, although there are a number of candidate immunonutrients/polyphenols with anti-inflammatory potential, their therapeutic use is hindered by a lack of understanding of their mechanism of action, particularly in primary human immune cells. Cellular metabolism has emerged as a major modulator of immune cell function, yet there has been limited study into the effects of polyphenols on immunometabolism. Here, we have investigated the activity of two plant-derived polyphenols, carnosol and curcumin, on the metabolism and downstream immune function of primary human DC. We demonstrate that the metabolic reprogramming which occurs in human DC upon LPS stimulation can be modulated by both carnosol and curcumin. We also demonstrate that these polyphenols regulate metabolic signaling through activation of AMPK and an associated inhibition of mTOR activity. Furthermore, we describe a novel relationship between AMPK signaling and induction of the immunomodulatory enzyme HO-1 by carnosol and curcumin. Together, this data demonstrates that regulation of metabolic signaling and function by naturally-derived polyphenols mediates their ability to promote tolerogenic DC.

While a number of studies have investigated metabolic reprogramming in activated murine DC, studies assessing human DC metabolism are comparatively scarce. LPS-stimulated BMDC have previously been observed to strongly upregulate aerobic glycolysis, and simultaneously downregulate oxidative phosphorylation via the action of iNOS-derived NO (10–12, 14). The results presented here demonstrate that human DC stimulated with LPS upregulate both glycolysis and oxidative phosphorylation within hours of activation. Furthermore, a transient increase in the glycolytic reserve and spare respiratory capacity (SRC) of human DC was observed within 6 h post-LPS stimulation, which was absent at 24 h post-LPS. Therefore, it can be ascertained that while human DC also display increased glycolytic metabolism after activation, unlike BMDC, they also upregulate oxidative phosphorylation. This disparity between murine and human DC is likely a result of their differing expression of iNOS, as human monocyte-derived DC do not readily express iNOS; however, some evidence suggests that certain human DC subsets can express iNOS *in vivo*, therefore the metabolic profile of these DC may differ from what is observed *in vitro* (17). Interestingly, a recent study by Basit et al. described differing metabolic programs employed by human DC subsets in response to stimulation with pRNA; plasmacytoid DC displayed an increase in oxidative phosphorylation whereas CD1c^+^ myeloid DC downregulated oxidative phosphorylation (33). Thus, it is important to consider that differences in the metabolism of DC may exist *in vivo* vs. *in vitro*, between DC subsets, or due to the type of stimulus employed. Further study of human DC under different conditions is required to delineate the impact of these variables on DC immunometabolism.

Consistent with the results presented here, Malinarich et al. have reported that monocyte-derived human DC matured with LPS are more glycolytic than immature DC, and do not downregulate oxidative phosphorylation (18). However, they also observed a reduced glycolytic reserve and SRC in mature compared to immature DC; a finding which, in fact, agrees with these results, as the metabolism of DC was assessed 24 h after maturation with LPS, by which time the increased glycolytic reserve and SRC observed in this study was absent. Interestingly, Everts et al. also observed an increase in the SRC of BMDC stimulated with LPS for 1 h, which was mediated by enhanced glycolytic flux into the Kreb's cycle (12). This increased flow of pyruvate into the Kreb's cycle was found to produce citrate necessary for *de novo* fatty acid synthesis in the maturing DC, providing lipids required to expand the endoplasmic reticulum and Golgi membranes in anticipation of increased protein production (12). Therefore, the transient increase in the glycolytic reserve and SRC of LPS-stimulated DC observed in this study may represent an early adaption of maturing DC to their new immunogenic functions, which is downregulated once adequate cellular remodeling has taken place. Meanwhile, the mature DC continues to display higher basal rates of glycolysis and oxidative phosphorylation to meet its increased energy demands. Thus, this study expands the current understanding of human DC metabolism, and also underscores the importance of accounting for temporal changes when analyzing the metabolism of immune cells.

The results of this study also further support our previous work which described the anti-inflammatory properties of the polyphenols, carnosol and curcumin, in human DC (19). The upregulation of glycolysis by BMDC in response to LPS has been demonstrated to promote their maturation, cytokine production and activation of T cells (10–12). Interestingly, DC treated with carnosol or curcumin displayed a reduced basal rate of glycolysis, and failed to upregulate their glycolytic reserve after 6 h of LPS stimulation. This reduced glycolytic flux was also manifest in the mitochondrial activity of carnosol- and curcumin-treated DC, as both polyphenols inhibited the increased SRC seen in response to LPS. Tolerogenic human DC have been reported to possess a greater capacity for oxidative phosphorylation and fatty acid oxidation, and are less glycolytic than mature DC (18). Therefore, it is possible that the anti-inflammatory effects of carnosol and curcumin in human DC are at least partly mediated by their inhibition of glycolysis, resulting in a diminished glycolytic reserve and SRC and failure to meet the bio-energetic requirements of maturation.

Both carnosol and curcumin have previously been reported to activate AMPK in skeletal muscle and cancer cell lines (34–37). In this study, carnosol and curcumin were found to activate AMPK in human DC. Furthermore, polyphenol-induced activation of AMPK resulted in the inhibition of mTOR activation in LPS-stimulated DC. We also demonstrate that AMPK activation by carnosol and curcumin is required to mediate their immunomodulatory effects in human DC given that pharmacological inhibition of AMPK can reverse the observed reduction of DC maturation by these polyphenols. In line with our study, Krawczyk et al. previously reported that AMPK signaling antagonizes the maturation of BMDC and inhibits their upregulation of glycolysis in response to LPS (10), while Carroll et al. found that AMPK-deficient BMDC display enhanced maturation and pro-inflammatory functions (13). Therefore, the activation of AMPK/inhibition of mTOR by carnosol and curcumin likely explains their regulation of DC metabolism and immune cell function observed in this study. Signaling via AMPK has previously been implicated in the upregulation of HO-1 by certain drugs (26, 27, 38), but there have been no such reports in human immune cells. Here, AMPK activation was found to upregulate expression of HO-1 in human DC, while inhibition of AMPK attenuated the induction of HO-1 by carnosol and curcumin. This study is therefore the first to report an association between AMPK signaling and HO-1 expression in human DC, and that the upregulation of HO-1 by carnosol and curcumin is at least partially dependent on their ability to activate AMPK. Indeed, a number of studies have identified cross-talk between AMPK and Nrf2, the major transcription factor in control of HO-1 expression (26, 38–40), hence it will be of interest to further explore the AMPK-Nrf2-HO-1 axis in the context of polyphenol-mediated immune modulation. Interestingly, a number of xenobiotics, including various polyphenols, have been reported to activate AMPK via an increase in the AMP:ATP ratio; this is achieved by inhibition of the mitochondrial electron transport chain complexes (41). Curcumin, in particular, has been shown to inhibit ATP synthase in mitochondrial preparations, thereby limiting ATP production and increasing the ratio of AMP to ATP (42). Given that a number of polyphenols also appear to inhibit ATP synthase or complex I (24, 43), it is likely that carnosol acts in a similar fashion. Therefore, elevation of AMP levels represents a probable mechanism by which carnosol and curcumin activate AMPK in human DC, however, further research is required to confirm this.

**Figure 6:**
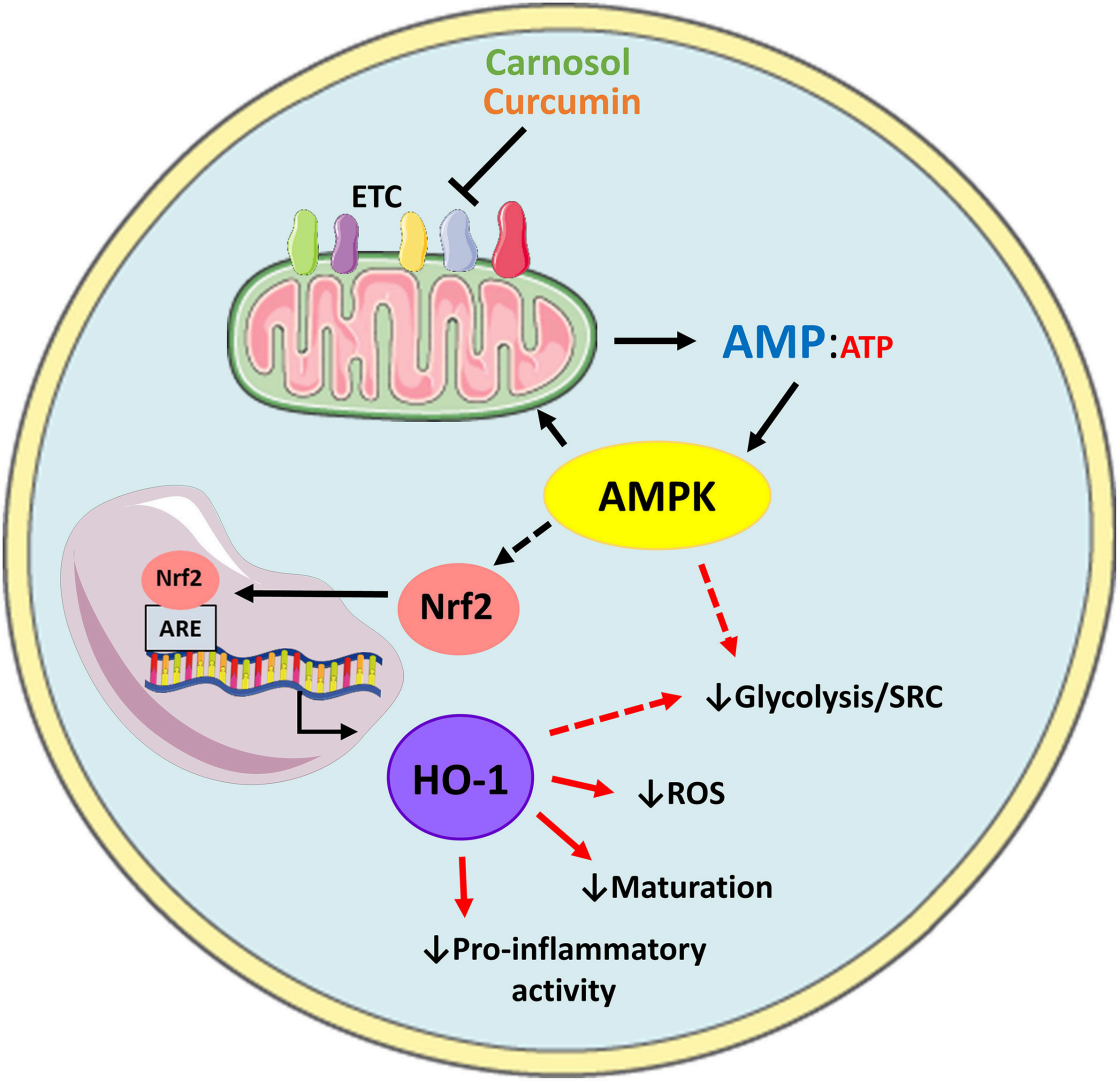
Proposed model of AMPK-dependent modulation of human DC metabolism and immune function by carnosol and curcumin. Carnosol and curcumin are polyphenols which have been shown to inhibit components of the ETC, resulting in reduced ATP production and elevated AMP levels. AMP activates AMPK, which results in downstream activation of Nrf2. Nrf2 translocates to the nucleus and induces transcription of HO-1. HO-1 and its products can then act as antioxidants to neutralize ROS produced by mitochondrial metabolism, and downregulate DC maturation and pro-inflammatory functions. Additionally, AMPK or HO-1 may mediate the reduced rate of glycolysis and SRC observed in carnosol- and curcumin-treated DC (red dashed arrows).

In conclusion, our data describes the metabolic changes arising from the activation of human DC, and characterizes a hitherto-unidentified role for the HO-1 system in immunometabolism. The data presented here supports a model whereby activation of AMPK by carnosol and curcumin leads to the upregulation of HO-1, which mediates the downstream immunomodulatory activity of these polyphenols in human DC (Figure 6). These results are also suggestive that the anti-inflammatory phenotype characteristic of immune cells with higher catabolic metabolism and AMPK signaling may arise from increased expression of HO-1, however future studies in HO-1 deficient cells are required to fully validate this hypothesis. Although our study supports the use of the polyphenols carnosol and curcumin as potential immunonutrient supplements, translation of these results to a clinical setting requires careful consideration regarding drug formulation and administration. One of the caveats associated with these polyphenols is their poor solubility in aqueous solutions, which may limit their bioavailability by certain routes of administration. Additionally, polyphenols have been described to undergo metabolic alterations during digestion via the intestinal microbiota, which could alter their metabolic and immunological properties as described here (44–46). Efforts made to improve the oral bioavailability of polyphenols such as curcumin, or to utilize alternative routes of administration, have been met with success in pre-clinical studies and clinical trials (47–50). It is hoped that future research can determine whether these polyphenols display similar effects on DC immunometabolism and function in an *in vivo* setting. Research into the use of polyphenols as clinically relevant immunonutrient supplements has expanded greatly over the last number of years and our data highlighting specific effects on key cells relevant to inflammatory and autoimmune disease provides further evidence attesting to their use as potential immune modulating compounds.

## Author Contributions

NC, JF, and AD conceptualized and designed experiments. NC and HF performed experiments. NC, HF, and AD wrote the manuscript.

### Conflict of Interest Statement

The authors declare that the research was conducted in the absence of any commercial or financial relationships that could be construed as a potential conflict of interest.
