# Supplementary material for: Plant-Derived Polyphenols Modulate Human Dendritic Cell Metabolism and Immune Function via AMPK-Dependent Induction of Heme Oxygenase-1
Source: Front Immunol. 2019 Mar 1;10:345. doi: 10.3389/fimmu.2019.00345 (PMC6405514; doi:10.3389/fimmu.2019.00345)
Supplement: Supplementary file 1 [file Data_Sheet_1.PDF]

Supplementary Material

Supplementary Figures

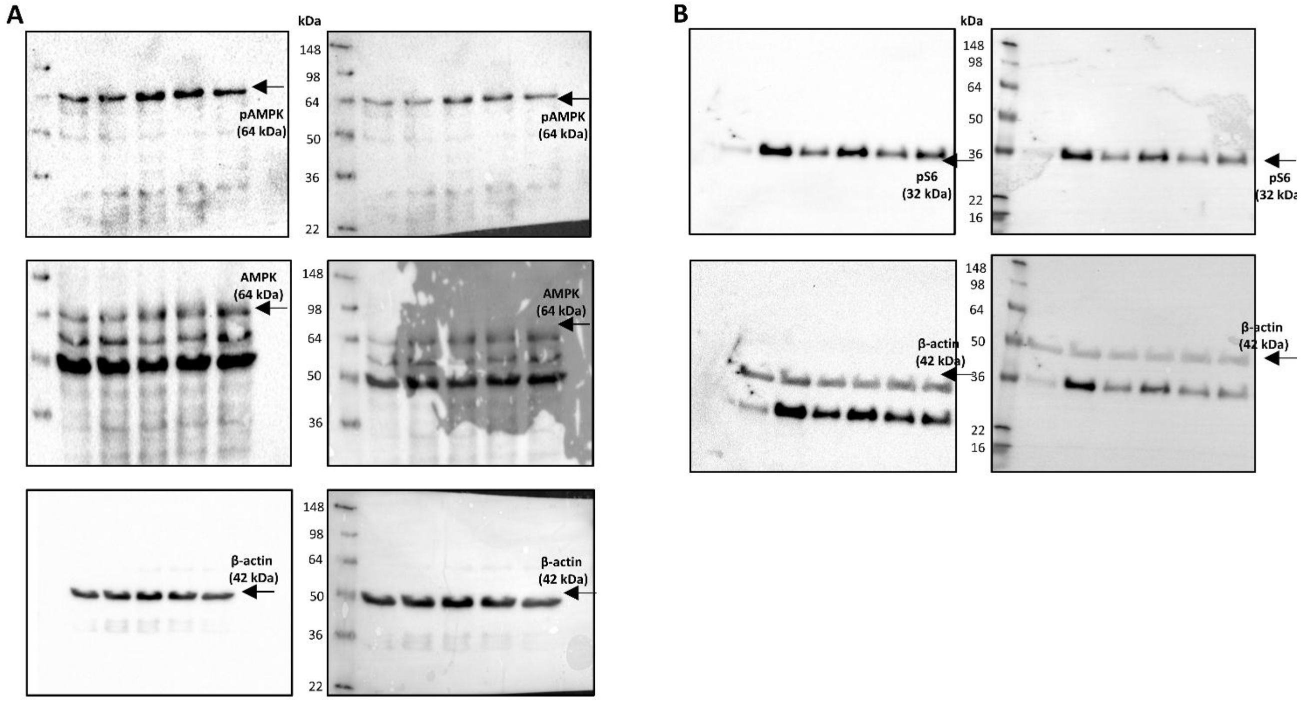

Supplementary Figure 1. Full length blots for Figure 3A and Figure 3C. AMPK and mTOR activation in human DC was measured by Western blot. Phospho-AMPK and total-AMPK blots were derived from separate gels. The phospho-AMPK and phospho-S6 membranes were then re-probed for β-actin as a loading control. Blots were developed using enhanced chemiluminescent substrate with a BioRad ChemiDoc MP system. Full scan images of the cropped exposure images used in Figure 3A **(A)** and Figure 3C **(B)** are provided alongside merged images of the exposed bands with the protein ladder to show molecular weights of the proteins.

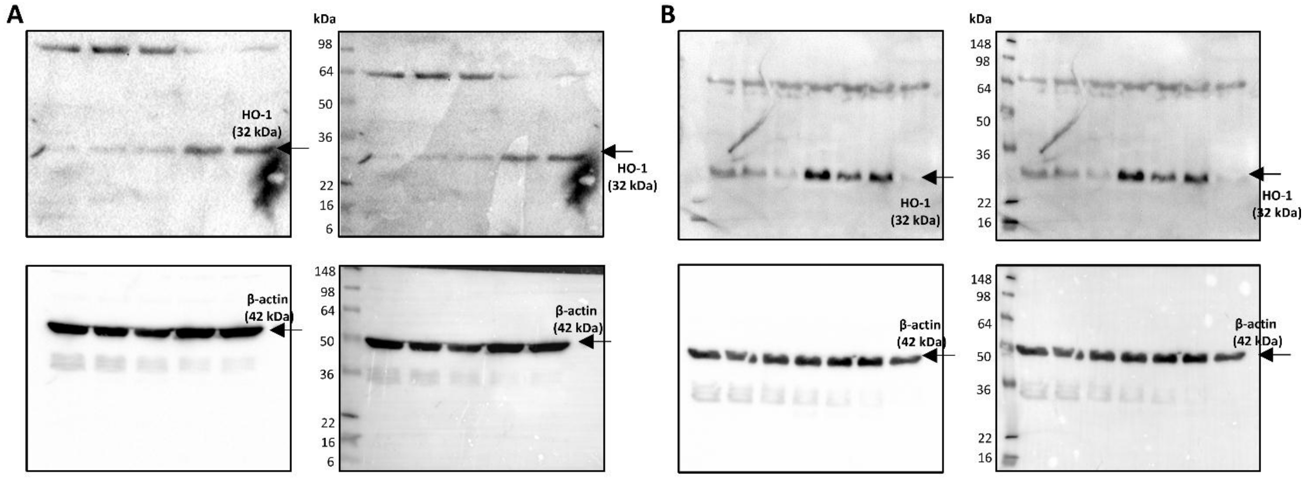

Supplementary Figure 2. Full length blots for Figure 3D and Figure 3E. HO-1 expression in human DC was measured by Western blot. Membranes were first probed for HO-1 and then re-probed for β-actin as a loading control. Blots were developed using enhanced chemiluminescent substrate with a BioRad ChemiDoc MP system. Full scan images of the cropped exposure images used in Figure 3D **(A)** and Figure 3E **(B)** are provided alongside merged images of the exposed bands with the protein ladder to show molecular weights of the proteins.

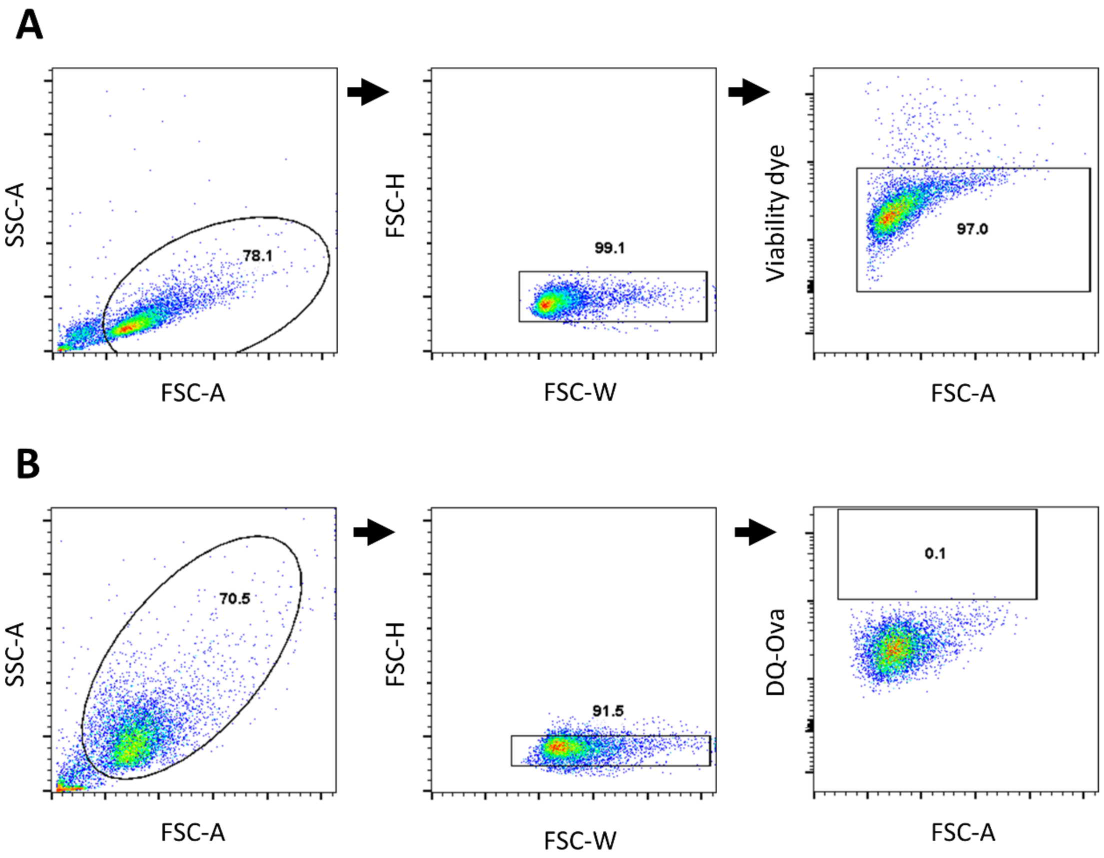

Supplementary Figure 3. Flow cytometry gating strategies. (A) To assess expression of maturation markers by DC, the DC population was first selected by FSC and SSC. Single cells were then gated on by FSC width and height. Viable cells were then selected on the basis of viability dye exclusion. **(B)** To measure DQ-Ova uptake, the DC population was first selected on the basis of FSC and SSC in order to exclude debris and dying cells. Single cells were then selected on the basis of FSC width and height. The DQ-Ova+ cell gate was then drawn using control cells which were not incubated with DQ-Ova.
